# Supplementary material for: Asynchronous magnetic resonance elastography: Shear wave speed reconstruction using noise correlation of incoherent waves
Source: Magn Reson Med. 2022 Oct 27;89(3):990–1001. doi: 10.1002/mrm.29502 (PMC9792433; doi:10.1002/mrm.29502)
Supplement: Supplementary file 1 — DATA S1 MATLAB code used to generate the numerical simulations of Figure 1 [file MRM-89-990-s002.zip › k-Wave/helpfiles/example_ivp_recording_particle_velocity.html]

Recording The Particle Velocity Example (k-Wave)


# Recording The Particle Velocity Example

This example demonstrates how to record the particle velocity using a Cartesian or binary sensor mask. It builds on the Homogeneous Propagation Medium and Heterogeneous Propagation Medium examples.

- Open the file in the MATLAB Editor
- Run the file in MATLAB

## Contents

- Setting the acoustic variables that are recorded
- Defining the time array
- Running the simulation

## Setting the acoustic variables that are recorded

By default, the first-order simulation functions in k-Wave return the pressure field recorded at each time step at the positions specified by `sensor.mask`. This data is assigned directly to the output argument `sensor_data` (unless using a sensor mask defined by opposing corners). It is also possible to control the acoustic variables that are recorded by the sensor mask by setting the value of `sensor.record`. The desired field parameters are listed as strings within a cell array. For example, to record both the acoustic pressure and the particle velocity, `sensor.record` should be set to `{'p', 'u'}`. If a value for `sensor.record` is set, the output `sensor_data` returned from the simulation is defined as a structure, with the recorded acoustic variables appended as structure fields. For example, if `sensor.record = {'p', 'p_max', 'u'}`, then the individual output variables are accessed as `sensor_data.p`, `sensor_data.p_max`, `sensor_data.ux`, `sensor_data.uy` (similarly for other dimensions). A full list of other `sensor.record` options is given in `kspaceFirstOrder2D`.

## Defining the time array

In the previous examples, `kWaveGrid` is used to define the properties of the spatial discretisation. After the `kWaveGrid` object is created, the only parameters that can be modified by the user are the number and size of the time steps used for the simulation. These are defined by the parameters `kgrid.Nt` and `kgrid.dt`. If either of these are set to `'auto'` (the default), the time array is automatically calculated within `kspaceFirstOrder2D` by calling the `makeTime` method of the `kWaveGrid` class. This sets the total time to the time it would take for an acoustic wave to travel across the longest grid diagonal at the minimum sound speed. The time step is based on a Courant-Friedrichs-Lewy (CFL) number of 0.3 and the maximum sound speed in the medium, where `kgrid.dt = CFL * dx_min / c0_max` (see the k-Wave manual for a detailed discussion on the relationship between the CFL number and accuracy and stability).

Instead of leaving `kgrid.Nt` and `kgrid.dt` set to `'auto'`, it is also possible to set the time array manually. There are two methods of the `kWaveGrid` class that can be used: (1) `makeTime`, which sets the time parameters based on the sound speed and CFL as described above, and (2) `setTime`, which is used to set `Nt` and `dt` directly. Here the time array is defined using `makeTime` using the default CFL and a simulation time of 6 us.

```
% create time array
t_end = 6e-6;       % [s]
kgrid.makeTime(kgrid, medium.sound_speed, [], t_end);
```

After creation, the number of time points and the size of the time step can be queried using `kgrid.Nt` and `kgrid.dt`, and the complete time array can be return using `kgrid.t_array`. Note, the time array is always evenly spaced and monotonically increasing.

## Running the simulation

The simulation is run in the same way as in previous examples. In this example, four sensor points are defined centered about the position of a small circular source, and the sensor is set to record both the acoustic pressure and the particle velocity. A plot of the initial pressure distribution and the sensor mask is given below.

```
% define four sensor points centered about source.p0
sensor_radius = 40; % [grid points]
sensor.mask = zeros(Nx, Ny);
sensor.mask(Nx/2 + sensor_radius, Ny/2) = 1;
sensor.mask(Nx/2 - sensor_radius, Ny/2) = 1;
sensor.mask(Nx/2, Ny/2 + sensor_radius) = 1;
sensor.mask(Nx/2, Ny/2 - sensor_radius) = 1;

% set the acoustic variables that are recorded
sensor.record = {'p', 'u'};

% run the simulation
sensor_data = kspaceFirstOrder2D(kgrid, medium, source, sensor);
```

The pressure and velocity signals recorded at the four sensor positions are shown below (these are indexed top to bottom, left to right). The recorded pressure signals are shown in the left column. As the sensor points are all equidistance from the center of the source, the signals appear the same. The recorded particle velocity signals in the x-direction are shown in the central column. The signals from the second and third sensor points are mirrored as the wave at these points is travelling in opposite directions. The signals at the first and fourth sensor points are almost zero because the wave at these positions is travelling largely in the y-direction. The recorded particle velocity signals in the y-direction are shown in the right column. The signals from the first and fourth sensor points are mirrored as the wave at these points is travelling in opposite directions. The signals at the second and third sensor points are almost zero because the wave at these positions is travelling largely in the x-direction.

Note, the first-order simulation functions use both spatially and temporally staggered grids. This means the output pressure and velocity values are offset from each other. For example, `sensor_data.ux` is obtained at grid points staggered in the x-direction by `+kgrid.dx/2` and in the temporal direction by `-kgrid.dt/2`. More information is given in the k-Wave Manual.
